# The Week in the Courts

**Published:** 1911-04-15

**Authors:** 


### The Week in the Courts.

### THE SALE OF POISONS BY SEEDSMEN.

In a King's Bench Divisional Court, before Justices Phillimore and Horridge, the Pharmaceutical Society appealed against a decision of the Lichfield County Court Judge in a case in which the society sued a seedsman named Jacks for a penalty of £5 under section 15 of the Pharmacy Act of 1868 for having sold a fumigator containing nicotine contrary to the provisions of the Act of 1868, as amended by the Poisons and Pharmacy Act 1908. Jacks holds a licence to sell horticultural and agricultural poisons, but in selling the article named above he omitted to label it with his name and address as required by regulations made by Order in Council. The County Court Judge held that the society could not recover under section 15 for this offence, and that proceedings should rather have been taken before a magistrate under section 17. The Divisional Court reversed this decision, and judgment with costs was entered for the society. The result of this judgment, which is of considerable importance to sellers of poison, is to establish that if a licensed dealer in horticultural and agricultural poisons fails to conform to the prescribed regulations as to labelling, etc., he is in the position of a person who does not hold a licence, and is liable to a penalty of £5 for selling poison as well as a penalty for failing to label it properly.

